# Supplementary material for: Associations of Preterm Birth with Dental and Gastrointestinal Diseases: Machine Learning Analysis Using National Health Insurance Data
Source: Int J Environ Res Public Health. 2023 Jan 18;20(3):1732. doi: 10.3390/ijerph20031732 (PMC9914760; doi:10.3390/ijerph20031732)
Supplement: Supplementary file 1 [file ijerph-20-01732-s001.zip › ijerph-2154604-SI.pdf]

Supplementary Table S1. ICD-10 code for preterm birth and dental/gastrointestinal disease

		Code	Description
PTB	PROM	O42.00	Onset of labor within 24 hours of rupture (0-33 weeks of gestation)
		O42.01	Onset of labor within 24 hours of rupture (34-36 weeks of gestation)
		O42.10	Onset of labor after 24 hours of rupture (0-33 weeks of gestation)
		O42.11	Onset of labor after 24 hours of rupture (34-36 weeks of gestation)
		O42.20	Prolonged labor because of treatment (0-33 weeks of gestation)
		O42.21	Prolonged labor because of treatment (34-36 weeks of gestation)
		O42.90	Unspecified length of time between rupture and onset of labor (0-33 weeks of
		O42.91	Unspecified length of time between rupture and onset of labor (34-36 weeks of
	PTL	O60.11	Preterm delivery with preterm labor (0-33 weeks of gestation)
		O60.12	Preterm delivery with preterm labor (34-36 weeks of gestation)
		O60.19	Preterm delivery with preterm labor (unspecified weeks of gestation)
	Other	O60.31	Preterm delivery without preterm labor (0-33 weeks of gestation)
		O60.32	Preterm delivery without preterm labor (34-36 weeks of gestation)
		O60.39	Preterm delivery without preterm labor (unspecified weeks of gestation)
Crohn		K50	Crohn's disease
GERD		K210	Gastroesophageal reflux disease
IBS		K58	Irritable bowel syndrome
UC		K51	Ulcerative colitis
Dental Cavity		K02	Dental cavity
Oral Mucositis		K12.3	Oral mucositis
Periodontitis		K052/0528/0529	Aggressive periodontitis
		K053/0538/0539,	Chronic periodontitis
Salivary Gland		K11	Salivary gland disease
Tooth Loss		K08.4	Tooth loss
Infertility		N97	Infertility

PTB Preterm Birth, PROM Premature Rupture of Membranes, PTL Preterm Labor with Preterm Delivery

Supplementary Table S2. ATC code for medication

Medication	Code	Description	
	N05BA	Benzodiazepine derivatives	(N05 Psychoanaleptics, N05B Anxiolytics)
Benzodiazepine	N05CD	Benzodiazepine derivatives	(N05 Psychoanaleptics, N05C Hypnotics and sedatives)
	N05CF	Benzodiazepine-related [illegible]	(N05 Psychoanaleptics, N05C Hypnotics and sedatives)
Calcium Channel [illegible]	C08	Calcium channel blockers	
Nitrate	C01DA	Organic nitrates	
Progesterone	G03	Sex hormones and modulators of the genital system	
Sleeping Pills	N05C	Hypnotics and sedatives	
Tricyclic [illegible]	N06A	Tricyclic Antidepressants	

Supplementary Table S3. Descriptive statistics

Variable	Term Birth (N = 117321)		Preterm Birth (PTB4) (N=7285)		P-Value
		Mean		*Mean*	
Age		31.81		32.10	<0.0001
Socioeconomic Status		11.08		11.11	0.1809
	Count	*%*	*Count*	*%*	
GERD_2002	759	0.65	50	0.69	0.6845
GERD_2003	953	0.81	72	0.99	0.1065
GERD_2004	1,313	1.12	89	1.22	0.4207
GERD_2005	1,705	1.45	137	1.88	0.0034
GERD_2006	2,233	1.90	165	2.26	0.0293
GERD_2007	3,263	2.78	231	3.17	0.0506
GERD_2008	3,952	3.37	304	4.17	0.0002
GERD_2009	5,616	4.79	411	5.64	0.0010
GERD_2010	6,646	5.66	468	6.42	0.0067
GERD_2011	7,777	6.63	559	7.67	0.0005
GERD_2012	8,921	7.60	598	8.21	0.0594
GERD_2013	9,351	7.97	651	8.94	0.0032
GERD_2014	10,697	9.12	711	9.76	0.0652
GERD_2015	11,296	9.63	767	10.53	0.0117
GERD_2016	11,788	10.05	855	11.74	<0.0001
Periodontitis_2002	48	0.04	2	0.03	0.5778
Periodontitis_2003	66	0.06	5	0.07	0.6675
Periodontitis_2004	120	0.10	6	0.08	0.6037
Periodontitis_2005	106	0.09	4	0.05	0.3230
Periodontitis_2006	160	0.14	14	0.19	0.2159
Periodontitis_2007	93	0.08	6	0.08	0.9276
Periodontitis_2008	69	0.06	15	0.21	<0.0001
Periodontitis_2009	120	0.10	5	0.07	0.3787
Periodontitis_2010	145	0.12	5	0.07	0.1893
Periodontitis_2011	75	0.06	5	0.07	0.8777
Periodontitis_2012	73	0.06	1	0.01	0.0992
Periodontitis_2013	90	0.08	6	0.08	0.8661
Periodontitis_2014	78	0.07	5	0.07	0.9450
Periodontitis_2015	108	0.09	2	0.03	0.0716
Periodontitis_2016	142	0.12	6	0.08	0.3524
Dental_Cavity_2002	173	0.15	9	0.12	0.6040
Dental_Cavity_2003	223	0.19	14	0.19	0.9682
Dental_Cavity_2004	207	0.18	27	0.37	0.0002
Dental_Cavity_2005	165	0.14	15	0.21	0.1547
Dental_Cavity_2006	170	0.14	12	0.16	0.6673
Dental_Cavity_2007	121	0.10	29	0.40	<0.0001
Dental_Cavity_2008	116	0.10	10	0.14	0.3171
Dental_Cavity_2009	133	0.11	8	0.11	0.9303
Dental_Cavity_2010	147	0.13	16	0.22	0.0307
Dental_Cavity_2011	56	0.05	3	0.04	0.8030
Dental_Cavity_2012	157	0.13	12	0.16	0.4868
Dental_Cavity_2013	249	0.21	8	0.11	0.0615
Dental_Cavity_2014	150	0.13	1	0.01	0.0066
Dental_Cavity_2015	42	0.04	1	0.01	0.3250
Dental_Cavity_2016	159	0.14	2	0.03	0.0127

Tooth_Loss_2002	4	0.00	0	0.00	0.6182
Tooth_Loss_2003	5	0.00	0	0.00	0.5774
Tooth_Loss_2004	4	0.00	0	0.00	0.6182
Tooth_Loss_2005	7	0.01	0	0.00	0.5097
Tooth_Loss_2006	4	0.00	0	0.00	0.6182
Tooth_Loss_2007	8	0.01	0	0.00	0.4809
Tooth_Loss_2008	2	0.00	1	0.01	0.0424
Tooth_Loss_2009	4	0.00	0	0.00	0.6182
Tooth_Loss_2010	11	0.01	1	0.01	0.7135
Tooth_Loss_2011	9	0.01	3	0.04	0.0047
Tooth_Loss_2012	1	0.00	0	0.00	0.8032
Tooth_Loss_2013	5	0.00	0	0.00	0.5774
Tooth_Loss_2014	8	0.01	0	0.00	0.4809
Tooth_Loss_2015	8	0.01	0	0.00	0.4809
Tooth_Loss_2016	2	0.00	1	0.01	0.0424
Salivary_Gland_2002	104	0.09	5	0.07	0.5751
Salivary_Gland_2003	141	0.12	11	0.15	0.4647
Salivary_Gland_2004	175	0.15	6	0.08	0.1463
Salivary_Gland_2005	166	0.14	14	0.19	0.2691
Salivary_Gland_2006	224	0.19	18	0.25	0.2908
Salivary_Gland_2007	273	0.23	18	0.25	0.8050
Salivary_Gland_2008	269	0.23	20	0.27	0.4359
Salivary_Gland_2009	337	0.29	30	0.41	0.0570
Salivary_Gland_2010	336	0.29	22	0.30	0.8093
Salivary_Gland_2011	394	0.34	23	0.32	0.7730
Salivary_Gland_2012	377	0.32	31	0.43	0.1309
Salivary_Gland_2013	473	0.40	24	0.33	0.3327
Salivary_Gland_2014	528	0.45	27	0.37	0.3232
Salivary_Gland_2015	420	0.36	29	0.40	0.5795
Salivary_Gland_2016	332	0.28	26	0.36	0.2528
Ulcerative_Colitis_2002	50	0.04	2	0.03	0.5386
Ulcerative_Colitis_2003	66	0.06	5	0.07	0.6675
Ulcerative_Colitis_2004	55	0.05	0	0.00	0.0645
Ulcerative_Colitis_2005	46	0.04	4	0.05	0.5162
Ulcerative_Colitis_2006	54	0.05	3	0.04	0.8511
Ulcerative_Colitis_2007	47	0.04	3	0.04	0.9631
Ulcerative_Colitis_2008	54	0.05	3	0.04	0.8511
Ulcerative_Colitis_2009	66	0.06	2	0.03	0.3071
Ulcerative_Colitis_2010	57	0.05	4	0.05	0.8129
Ulcerative_Colitis_2011	78	0.07	6	0.08	0.6124
Ulcerative_Colitis_2012	90	0.08	3	0.04	0.2812
Ulcerative_Colitis_2013	83	0.07	4	0.05	0.6195

Ulcerative_Colitis_2014	100	0.09	3	0.04	0.2042
Ulcerative_Colitis_2015	118	0.10	4	0.05	0.2265
Ulcerative_Colitis_2016	108	0.09	4	0.05	0.3046
Crohn_2002	122	0.10	12	0.16	0.1249
Crohn_2003	92	0.08	8	0.11	0.3585
Crohn_2004	105	0.09	6	0.08	0.8429
Crohn_2005	86	0.07	4	0.05	0.5706
Crohn_2006	65	0.06	7	0.10	0.1609
Crohn_2007	47	0.04	6	0.08	0.0893
Crohn_2008	35	0.03	3	0.04	0.5904
Crohn_2009	36	0.03	2	0.03	0.8782
Crohn_2010	41	0.03	2	0.03	0.7383
Crohn_2011	48	0.04	1	0.01	0.2561
Crohn_2012	56	0.05	2	0.03	0.4362
Crohn_2013	37	0.03	2	0.03	0.8484
Crohn_2014	49	0.04	3	0.04	0.9811
Crohn_2015	53	0.05	3	0.04	0.8760
Crohn_2016	55	0.05	2	0.03	0.4518
IBS_2002	2,114	1.80	156	2.14	0.0355
IBS_2003	2,382	2.03	158	2.17	0.4169
IBS_2004	2,824	2.41	211	2.90	0.0086
IBS_2005	3,391	2.89	263	3.61	0.0004
IBS_2006	3,669	3.13	269	3.69	0.0075
IBS_2007	869	0.74	65	0.89	0.1456
IBS_2008	149	0.13	14	0.19	0.1353
IBS_2009	143	0.12	17	0.23	0.0099
IBS_2010	129	0.11	10	0.14	0.4980
IBS_2011	59	0.05	6	0.08	0.2447
IBS_2012	49	0.04	4	0.05	0.5976
IBS_2013	39	0.03	5	0.07	0.1187
IBS_2014	38	0.03	3	0.04	0.6881
IBS_2015	37	0.03	4	0.05	0.2859
IBS_2016	32	0.03	2	0.03	0.9929
Infertility_2002	22	0.02	2	0.03	0.6035
Infertility_2003	35	0.03	6	0.08	0.0165
Infertility_2004	50	0.04	4	0.05	0.6248
Infertility_2005	86	0.07	7	0.10	0.4896
Infertility_2006	133	0.11	16	0.22	0.0109
Infertility_2007	179	0.15	20	0.27	0.0114
Infertility_2008	257	0.22	38	0.52	<0.0001
Infertility_2009	330	0.28	30	0.41	0.0440
Infertility_2010	516	0.44	54	0.74	0.0002
Infertility_2011	839	0.72	92	1.26	<0.0001
Infertility_2012	1,295	1.10	143	1.96	<0.0001
Infertility_2013	2,275	1.94	255	3.50	<0.0001
Infertility_2014	4,907	4.18	501	6.88	<0.0001
Infertility_2015	10,541	8.98	1,072	14.72	<0.0001
Infertility_2016	21,273	18.13	2,015	27.66	<0.0001
Benzodiazepine	47,580	40.56	3,203	43.97	<0.0001
CCB	154	0.13	10	0.14	0.8909
Nitrate	194	0.17	15	0.21	0.4119

Progesterone	18,580	15.84	1,382	18.97	<0.0001
Sleeping Pill	5,535	4.72	447	6.14	<0.0001
Tricyclic Antidepressant	11,254	9.59	787	10.80	0.0007
Oral_Mucositis_2012	61	0.05	4	0.05	0.9158
Oral_Mucositis_2013	70	0.06	3	0.04	0.5269
Oral_Mucositis_2014	101	0.09	4	0.05	0.3735
Oral_Mucositis_2015	91	0.08	6	0.08	0.8867
Oral_Mucositis_2016	99	0.08	8	0.11	0.4721

GERD gastroesophageal reflux disease, IBS irritable bowel syndrome, CCB calcium channel blocker

Supplementary Table S4. Random forest variable importance - no sampling

	PTB1		PTB2		PTB3		PTB4	
1	SES	0.2	SES	0.2	SES	0.28	SES	0.2
2	Age	0.2	Age	0.2	Age	0.23	Age	0.2
3	GERD_201	0.0	Infertility_2	0.0	GERD_20	0.02	GERD_20	0.0
4	GERD_201	0.0	Benzodiaze	0.0	GERD_20	0.02	GERD_20	0.0
5	GERD_201	0.0	GERD_201	0.0	Progestero	0.02	GERD_20	0.0
6	Progestero	0.0	GERD_201	0.0	GERD_20	0.02	Progestero	0.0
7	TCA	0.0	GERD_201	0.0	GERD_20	0.02	GERD_20	0.0
8	GERD_201	0.0	GERD_201	0.0	GERD_20	0.01	GERD_201	0.0
9	Benzodiaze	0.0	GERD_201	0.0	Benzodiaz	0.02	TCA	0.0
10	GERD_201	0.0	GERD_201	0.0	GERD_20	0.02	Infertility_	0.0
11	GERD_201	0.0	Infertility_2	0.0	GERD_20	0.01	GERD_20	0.0
12	Infertility_	0.0	GERD_201	0.0	Infertility_	0.01	GERD_20	0.0
13	GERD_200	0.0	Progesteron	0.0	TCA	0.02	Benzodiaze	0.0
14	GERD_200	0.0	TCA	0.0	GERD_20	0.01	GERD_20	0.0
15	GERD_201	0.0	GERD_200	0.0	Infertility_	0.01	Infertility_	0.0
16	IBS_2006	0.0	GERD_200	0.0	GERD_20	0.01	GERD_20	0.0
17	GERD_200	0.0	GERD_200	0.0	IBS_2005	0.01	GERD_20	0.0
18	IBS_2005	0.0	IBS_2005	0.0	GERD_20	0.01	IBS_2006	0.0
19	Infertility_	0.0	IBS_2006	0.0	IBS_2002	0.00	GERD_20	0.0
20	GERD_200	0.0	Sleeping	0.0	IBS_2003	0.00	IBS_2004	0.0
21	IBS_2004	0.0	IBS_2004	0.0	IBS_2004	0.01	IBS_2005	0.0
22	Infertility_	0.0	Infertility_2	0.0	IBS_2006	0.01	IBS_2002	0.0
23	Sleeping	0.0	IBS_2002	0.0	Infertility_	0.01	IBS_2003	0.0
24	IBS_2002	0.0	IBS_2003	0.0	Sleeping	0.01	Infertility_	0.0
25	IBS_2003	0.0	Infertility_2	0.0	GERD_20	0.00	Sleeping	0.0
26	GERD_200	0.0	GERD_200	0.0	GERD_20	0.00	GERD_20	0.0
27	GERD_200	0.0	GERD_200	0.0	GERD_20	0.00	GERD_20	0.0
28	Infertility_	0.0	GERD_200	0.0	Infertility_	0.00	Infertility_	0.0
29	GERD_200	0.0	Infertility_2	0.0	GERD_20	0.00	GERD_20	0.0
30	GERD_200	0.0	GERD_200	0.0	GERD_20	0.00	GERD_20	0.0

GERD = gastroesophageal reflux disease, IBS = irritable bowel syndrome, SES = Socioeconomic Status, TCA = Tricyclic Antidepressant
